# Early Outcome, Cosmetic Result and Tolerability of an IOERT-Boost Prior to Adjuvant Whole-Breast Irradiation

**DOI:** 10.3390/cancers14153636

**Published:** 2022-07-26

**Authors:** Danny Jazmati, Edwin Bölke, Kati Halfmann, Bálint Tamaskovics, Eugen Ruckhäberle, Tanja Fehm, Jürgen Hoffmann, David Krug, Carolin Nestle Krämling, Stefanie Corradini, Wilfried Budach, Svjetlana Mohrmann, Jan Haussmann, Christiane Matuschek

**Affiliations:** 1 Department of Radiation Oncology, Heinrich Heine University, 40225 Duesseldorf, Germany; danny.jazmati@med.uni-duesseldorf.de (D.J.); kati.halfmann@med.uni-duesseldorf.de (K.H.); balint.tamaskovics@med.uni-duesseldorf.de (B.T.); wilfried.budach@med.uni-duesseldorf.de (W.B.); jan.haussmann@med.uni-duesseldorf.de (J.H.); 2 Department of Gynecology & Obstetrics, Heinrich-Heine University Dusseldorf, 40225 Duesseldorf, Germany; eugen.ruckhaeberle@med.uni-duesseldorf.de (E.R.); tanja.fehm@med.uni-duesseldorf.de (T.F.); juergen.hoffmann@med.uni-duesseldorf.de (J.H.); svejetlana.mohrmann@med.uni-duesseldorf.de (S.M.); 3 Department of Radiation Oncology, University Medical Center Schleswig-Holstein, 24105 Kiel, Germany; david.krug@uksh.de; 4 Department of Gynecology, Obstetrics and Senology, EVK, 40225 Duesseldorf, Germany; carolin.nestle-kraemling@evk-duesseldorf.de; 5 Department of Radiation Oncology, University Hospital, LMU Munich, 80539 Munich, Germany; stefanie.corradini@med.uni-muenchen.de

**Keywords:** breast cancer, radiation therapy, clinical outcome, skin reaction, LENT-SOMA

## Abstract

**Simple Summary:**

Our study with 139 breast cancer patients treated with intraoperative radiotherapy reports favorable data on the cosmetic outcome as well as the acute and early long-term side effects. Our oncologic control rates are comparable to the previous literature.

**Abstract:**

**Background/Aims:** Due to its favorable dose distribution and targeting of the region at highest risk of recurrence due to direct visualization of tumor bed, intraoperative electron radiation therapy (IOERT) is used as part of a breast-conserving treatment approach. The aim of this study was to analyze tumor control and survival, as well as the toxicity profile, and cosmetic outcomes in patients irradiated with an IOERT boost for breast cancer. **Materials and Methods**: 139 Patients treated at our institution between January 2010 and January 2015 with a single boost dose of 10 Gy to the tumor bed during breast-conserving surgery followed by whole-breast irradiation were retrospectively analyzed. **Results**: 139 patients were included in this analysis. The median age was 54 years (range 28–83 years). The preferred surgical strategy was segmental resection with sentinel lymphonodectomy (66.5%) or axillary dissection (23.1%). Regarding adjuvant radiotherapy, the vast majority received 5 × 1.8 Gy to 50.4 Gy. At a median follow-up of 33.6 months, recurrence-free and overall survival were 95.5% and 94.9%, respectively. No patient developed an in-field recurrence. Seven patients (5.0%) died during the follow-up period, including two patients due to disease recurrence (non-in-field). High-grade (CTCAE > 2) perioperative adverse events attributable to IOERT included wound healing disorder (N = 1) and hematoma (N = 1). High-grade late adverse events (LENT-SOMA grade III) were reported only in one patient with fat necrosis. Low-grade late adverse events (LENT-SOMA grade I-II) included pain (18.0%), edema (10.5%), fibrosis (21%), telangiectasia (4.5%) and pigmentation change (23.0%). The mean breast retraction assessment score was 1.66 (0–6). Both patients and specialists rated the cosmetic result “excellent/good” in 84.8% and 87.9%, respectively. **Conclusion**: Our study reports favorable data on the cosmetic outcome as well as the acute and early long-term tolerability for patients treated with an IOERT boost. Our oncologic control rates are comparable to the previous literature. However, prospective investigations on the role of IOERT in comparison to other boost procedures would be desirable.

## 1. Introduction

Breast cancer is the most common malignancy in women [1]. The multimodal treatment approach for localized non-metastatic disease is composed of surgery, radiotherapy and systemic treatment. An established standard for local treatment is breast-conserving surgery followed by adjuvant whole-breast irradiation [2]. Pathologic studies have revealed that relapses are most likely to occur in the vicinity of the surgical bed [3]. Consistently, randomized trials demonstrated that a further increase in dose (boost) to the lumpectomy bed can significantly improve local tumor control [4,5,6]. However, an improvement in survival was not found [4,7]. Multiple technical approaches are available for boost application. In addition to external irradiation, interstitial brachytherapy and intraoperative radiotherapy with electrons (IOERT) or photons (IORT) are established concepts. The one-off, targeted intraoperative radiation (TARGIT)—with Zeiss Intrabeam 600—directly after tumor removal is not inferior to external radiation therapy (EBRT) in terms of effectiveness [8,9]. Breast cancer brachytherapy is a proven treatment method. It enables precise postoperative radiation treatment after breast-conserving removal of the cancerous tumor [10]. This applies to dose escalation after homogeneous breast irradiation, but also to the sole radiotherapeutic modality (partial breast irradiation and for accelerated partial breast irradiation) in specially selected patients [10,11].

Regarding IOERT, a single intraoperative irradiation with electrons precedes adjuvant whole-breast radiotherapy (WBRT). IOERT is performed under direct visualization of the tumor bed. Since irradiation is performed immediately during surgery [12], residual tumor cells will be eliminated or at least its number reduced [13]. Considering the low alpha/beta values reported in breast cancer, the high single dose used in IOERT is theoretically of biological advantage [14]. In addition, better sparing of the skin can be achieved. With excellent oncologic control rates and minimal complications, early clinical data demonstrated the efficacy and safety [15,16].

Although IOERT is increasingly used for boost application, relatively few data have been published previously [17]. Given the very high single doses applied, further clinical experience regarding late effects is of major relevance. With improved survival rates, cosmetic outcome is becoming more relevant and continues to be a field for further investigations.

Therefore, we report our institutional experience concerning early outcome, toxicity and cosmetic results in patients with breast cancer receiving IOERT as a boost.

## 2. Materials and Methods

### 2.1. Patient Selection

**Figure 1:**
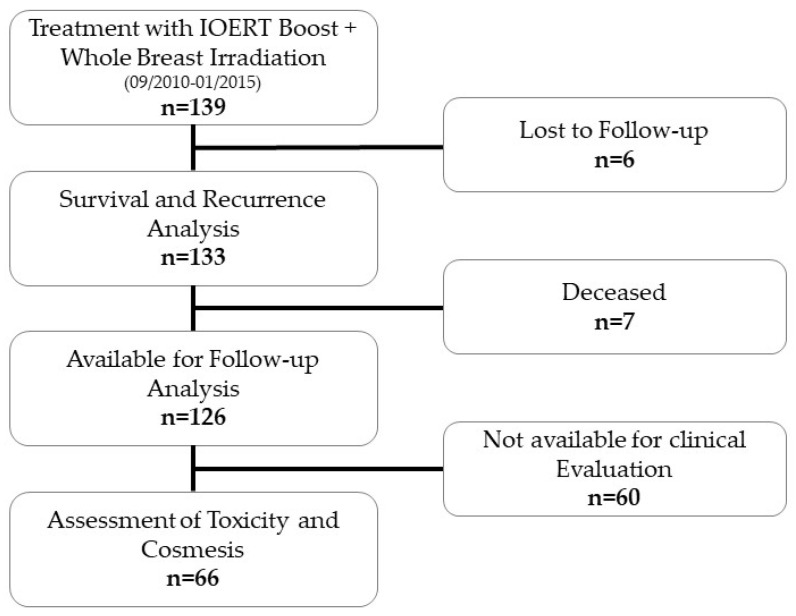
The Flow Diagram of our study.

Patients with histologically confirmed breast cancer receiving IOERT during breast-conserving surgery as a boost followed by WBRT were included in this investigation; 139 consecutive patients treated between January 2010 and January 2015 at our Breast Cancer Center Duesseldorf were included (Figure 1). Patients were selected according to S3-guidelines for breast cancer: indication for BCS, premenopausal patients or postmenopausal patients with tumor size ≥ 2 cm, G3, Her2 positive, triple negative, extensive intraductal component (EIC). Some patients received the boost as an individual decision, especially in patients with a tumor size of nearly 2 cm. The study was approved by the Ethical Board of the Heinrich Heine University Duesseldorf (Ref. nr. 4671).

### 2.2. Treatment

**Figure 2:**
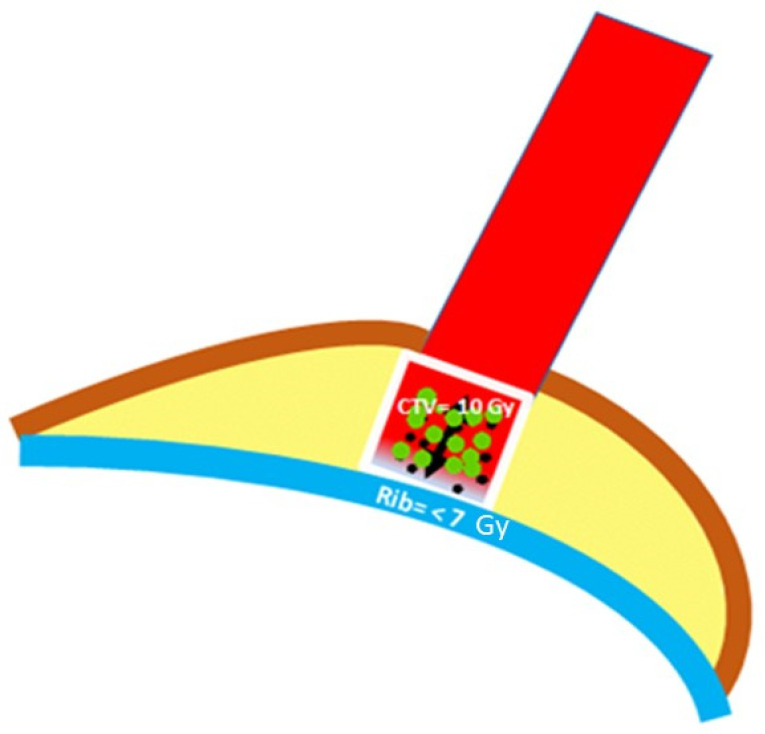
Schematic representation of the IOERT-situs in the breast: blue: rib; brown: skin; yellow: breast parenchyma; white frame, red inside with green dots: tumor bed = clinical target volume (CTV); red: tubus. Before starting the radiation, the distance between surface of the tumor bed and the rib will be measured using intraoperative ultrasound (black arrow). The prescribed single dose is 10 Gy to the tumor bed (red isodose). The dose constraint for the rib is a maximum of 7 Gy (purple isodose). The skin is outside the tubus.

**Figure 3:**
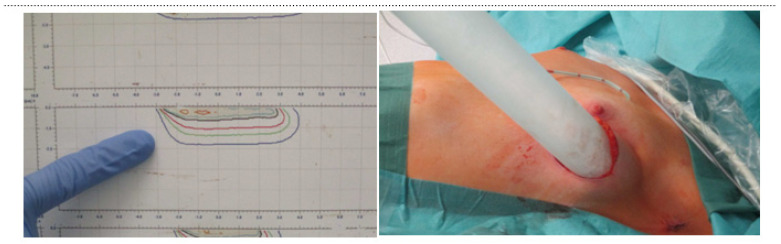
Plan and application of an IOERT.

For all patients, the overall treatment strategy was applied according to the national guidelines or international study protocols and determined in the context of an interdisciplinary tumor board. IOERT was recommended as part of a multidisciplinary treatment approach consisting of surgery, radiotherapy and systemic treatment. Depending on the individual risk profile, surgery was performed either up-front or after neoadjuvant therapy. In the case of neoadjuvant therapy, sentinel lymph node biopsy was performed prior to the initiation of systemic treatment. The standard surgical approach consisted of segmental resection with sentinel lymph node biopsy or axillary dissection. The size of the tumor bed and the distance of the tumor bed to rib and fascia were measured through intraoperative ultrasound. IOERT was performed using a dedicated mobile robotic linear accelerator (NOVAC 7, New Radiant Technology, Aprilia, Italy). Depending on the individual intraoperative anatomic situation, the tumor diameter, the thoracic wall thickness and the distance to the OAR rib were measured by intraoperative ultrasonography (Figure 2). The physician-physicist team selected the “dose plan of the day” including the tube diameter (40 or 50 mm), the tube angle (0°, 22.5° or 45°) and the electron beam energy (5, 7 or 9 MeV). A total dose of 10 Gy was administered to the 90% isodose using 5, 7 or 9 MeV electrons depending on the thickness of breast parenchyma and the distance to the rib. At the rib surface, a maximum dose of 7 Gy was allowed to avoid necrosis. We used this regimen according to the experience of other research groups [5]. Shielding disks were not necessary with this approach. In order to limit the dose applied to the ribs, compromises were allowed. All patients received adjuvant WBRT. Thereby, the entire breast was considered the clinical target volume (CTV). The planning target volume (PTV) was defined as the CTV with a margin of 7–10 mm. Figure 3 illustrates the plan and procedure. Depending on the individual risk profile, systemic treatment with chemotherapy, targeted and or endocrine therapy was given according to national guidelines.

### 2.3. Follow Up

Patient data, including oncological outcome and toxicity, were recorded at baseline, during treatment and at the follow-up (FU) visit. Patients were assessed at least weekly during adjuvant WBRT and followed up after completion of treatment according to national guidelines. All patients were offered follow-up care by the interdisciplinary treatment team consisting of gynecologists and radiation oncologists. Acute side effects were graded according to the National Cancer Institute Common Terminology Criteria for Adverse Events (CTCAE), version 4.0, grading system. Late effects were graded according to the late effects on normal tissues, in subjective, objective, management and analytic categories (LENT-SOMA). All side effects detected during and up to three months after the end of radiotherapy were defined as acute effects. Side effects occurring three months or more after completion of radiotherapy were considered as late side effects. At the time of data collection, all surviving patients were invited to participate in an analysis of cosmetic outcomes and late effects. In case of relapse, the imaging at the time of the local relapse was co-registered with the planning-CT scan and the treatment plan. The tubus position, tubus size, tubus angle and the energy were superimposed to determine the location of the relapse. Relapses were categorized as in-field or out-of-field failures.

### 2.4. Cosmetic Outcome

Of all survivors, 66 patients presented for cosmetic evaluation including breast retraction score and optical evaluation.

**Figure 4:**
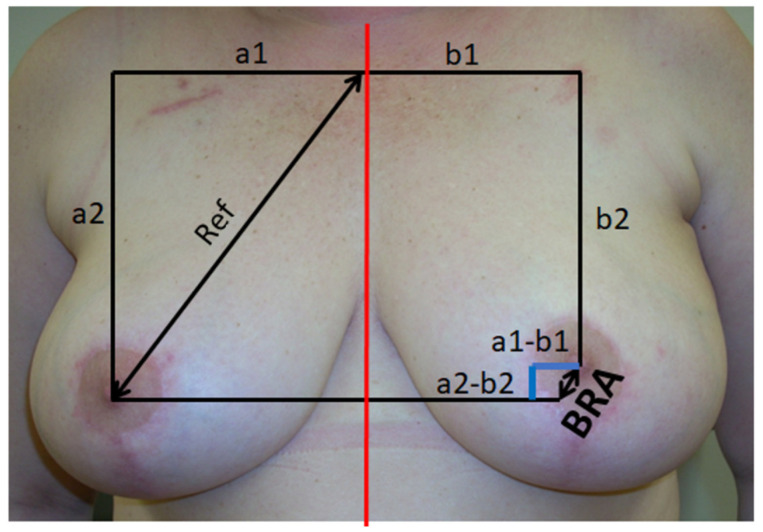
Breast retraction assessment (BRA): BRA scoring is used to assess asymmetry of the breast. A score of 0 represents the best result (no asymmetry). This patient, for example, has a low score < 2 (minimal asymmetry).

As described by Vrieland et al. [18] and in our previous work [19] the breast retraction score (BRA) was calculated to evaluate breast asymmetry (Figure 4). Thereby, a score of 0 constitutes the best outcome, while an increasing degree of impairment is found at higher values of the score.

The cosmetic results were independently graded by the patient and a radiation oncologist specialized for breast cancer. For better comparison to other series, we transformed our own score “very good”, “good”, “moderate”, “ moderate bad”, “bad”, “very bad” into the commonly used Harvard scale using four categories (“excellent”, “good”, “fair” and “poor”) [20].

### 2.5. Statistical Analysis

Qualitative data were presented as frequency (minimum-maximum) and percentage. Cut-off were based on known cut-off or median. The influence of different clinical and treatment variables on the cosmetic outcome was assessed using chi-square-test, Spearman’s correlation and Mann–Whitney U test. Recurrence was used to describe any form of relapse (local, regional and distant). Freedom from recurrence was defined as the absence of any recurrence from the date of surgery. Overall survival represents survival from diagnosis until death from any cause. Overall survival and recurrence-free survival were analyzed using the Kaplan–Meier method. Patients were censored if they were alive and free from recurrence at last follow-up. Log-rank test was used to compare survival. Statistical analyses were done in 5% alpha risk or 95% confidence interval using Statistical Package for the Social Sciences (SPSS) version 25.0 on Apple^®^.

## 3. Results

### 3.1. Baseline Characteristics

A total of 139 subjects with a median age of 54 years (range 28–83 years) were evaluable. The cohort included 77.0% with ductal carcinoma and 15.4% with lobular carcinoma. Anatomically, 40.6% of the cases were in the upper outer quadrant, 9.8% in the lower outer quadrant, 7.7% in the upper inner quadrant and 7% in the lower inner quadrant. At baseline, patients presented with comorbidities of the cardiovascular system (15.1%), lung (5.8%), thyroid (8.6%), immune system (5.0%), psyche (3.5%) and kidney (2.0%). Overall, 6.5% of the patients were active smokers. A family history for gynecological cancer was found in 27.3% of the patients. Of these, 43.6% had first-grade and 56.4% had second-grade relatives with gynecological cancer. In 10.8% of cases, re-resection was required.

Table 1 shows the tumor characteristics of all patients.

### 3.2. Treatment Data

#### 3.2.1. Surgery

The most common surgical approach was segmental resection with sentinel lymph node biopsy (66.5%) or axillary dissection (23.1%). Resection of the nipple-areolar complex was carried out in 4.2% of the patients due to tumor involvement.

#### 3.2.2. Radiotherapy

Regarding adjuvant radiotherapy, standard treatment consisted of 5 × 1.8 Gy to 50.4 Gy. One patient underwent moderate hypofractionation, one patient discontinued radiotherapy and another patient received dose reduction of WBRT in the presence of plasmacytoma. Nine patients received regional nodal irradiation of the infraclavicular and supraclavicular lymph nodes in addition to WBRT.

#### 3.2.3. Chemotherapy

Chemotherapy was administered either in a neoadjuvant (N = 19) or adjuvant (N = 60) setting in 79 patients. The combination of docetaxel, epirubicin and cyclophosphamide (TAC three weeks) was chosen most frequently as treatment in the neoadjuvant setting and a combination of docetaxel, 5-fluorouracil, epirubicin and cyclophosphamide (three cycles FEC q three weeks followed by Docetaxel q three weeks) in the adjuvant setting. Table 2 shows the chemotherapy regimes in our study.

#### 3.2.4. Endocrine and Targeted Therapy

In the context of clinical trials, ten patients received three weeks of endocrine induction with anastrozole, letrozole, or tamoxifen. All patients with HER2 overexpression received HER2-targeted therapy for a total of one year.

#### 3.2.5. Acute Toxicity

IOERT was found to be well tolerated. Only two patients presented with high-grade complications (CTCAE > grade 2) that could be attributable to the irradiation. One patient experienced impaired wound healing requiring secondary suture and the other patient developed a severe hematoma requiring axillary revision.

#### 3.2.6. Late Toxicity

Low-grade toxicities (LENT-SOMA Grade I-II) included pain (18.0%), edema (10.5%), fibrosis (21.0%), telangiectasia (4.5%) and pigmentation change (23.0%). One patient developed fat necrosis, likely unrelated to IOERT.

Table 3 shows the evaluation of the LENT-SOMA late toxicity.

### 3.3. Cosmetic Outcome

The mean BRA score was 1.66 (0–6). The cosmetic result was rated as excellent or good by 84.8% of patients. (excellent: 46.9%, good 37.9%, fair 12.1% and poor 3%). However, on average, the experts rated the cosmetic slightly better than the patients (excellent: 53%, good 34.8%, fair 7.6% and poor 1.5%). Thus, according to the expert rating, 87.8% of patients achieved an excellent or good result. Table 4 depicts the cosmetic outcome of all patients.

### 3.4. Predictors of Unsatisfactory Cosmetic Outcome

More advanced age (*p* = 0.001), greater BRA score (*p* < 0.001), higher degree of retraction (*p* = 0.005) and electron energy of 7 MeV compared to 5 MeV (*p* = 0.014) significantly correlated with poorer cosmetic outcome, assessed by the specialist. On the other hand, chemotherapy (*p* = 0.493), tumor stage (*p* = 0.126), tumor depth (*p* = 0.376), localization of primary (*p* = 0.344), re-resection (*p* = 0.179) or axillary dissection (*p* = 0.864) showed no significant correlation.

### 3.5. Oncological Outcome

At a median follow-up of 33.6 months, recurrence-free and overall survival were 95.5% and 94.9%, respectively. Of 139 patients, no patient developed in-field recurrence. However, four patients developed out-field recurrences in the ipsilateral breast 13, 23, 39 and 46 months postoperatively. Two patients developed contralateral breast cancer 34 and 58 months after surgery. Three patients were diagnosed with distant metastasis during follow-up at 17, 33 and 40 months after surgery. A total of seven patients were deceased during the observation period, including four due to disease recurrence. The nodal status had a significant effect on patient survival (*p* = 0.048). Median survival for negative nodal status was 72 months (95% CI: 70.32; 74.2) and for positive nodal status 62 months (95% CI: 56.36; 67.52).

Age and receptor status (any receptor positive/triple negative) also had a significant effect on the time of relapse occurrence: patients under 45 years of age developed a relapse after a mean of 63.4 months (95% CI: 56.76; 68.25) (Log Rank X2(1) = 4.95 *p* = 0.026) and patients with triple negative carcinoma after 58 months (95% CI: 49.5; 66.6); Log Rank X2(1) = 8.3; *p* = 0.004). The oncological outcome is shown in Table 5.

## 4. Discussion

This study demonstrated excellent oncologic results accompanied by good tolerability, minor early and late toxicity and satisfactory cosmetic outcome [8,9].

After a 3-year follow-up, the in-breast local progression-free survival and overall survival rates were 98.1% and 97.5%, respectively. No in-field recurrence occurred in the present study after a median follow-up of 33.6 months. Our cohort and treatment approach was consistent with the previous published literature. The largest study conducted on IOERT boost in breast cancer reported a local tumor control rate of 99.2% after 72.4 months [21,22]. It included 1109 patients who received an IOERT boost of 10 Gy, followed by WBRT with 50–54 Gy. Similarly, in a mono-institutional study compromising of 157 patients treated with an IOERT boost (10 Gy) prior to WBRT, the Heidelberg group described comparable oncological results [23]. In a single institutional randomized phase III study including patients with early stage breast cancer, Ciabattoni et al. demonstrated, in a comparison of 10 Gy IOERT boost or an external boost of 10 Gy in five fractions, comparable oncologic control rates and similar tolerability [13]. However, an IOERT boost was associated with an advantage in cosmesis, though this was influenced by baseline.

In the previously published literature, the most common perioperative complications after IOERT were hematoma (1.3–11.9%), wound healing disorder (1.3–7.0%) and infection (1.3–8.5%) [24,25,26,27]. Our data describing one patient with hematoma and one patient with wound healing disorder are thus consistent with previous analyses indicating a good perioperative tolerability.

Telangiectasia corresponds to a radiation-induced permanent dilatation of the superficial capillaries, which may have an impact on the cosmetic outcome. In the Lyon trial, the role of a 10 Gy external electron boost was investigated in a randomized fashion in 1024 patients. There was an increased risk with regard to telangiectasia in the boost group (12.4% vs. 5.9%) [5]. However, the results of the Lyon trial should be interpreted with caution, due to the distinct dose distribution of electrons. Since the skin, as an organ at risk for telangiectasia, can be almost completely spared, IOERT represents an attractive therapeutic procedure. The results of an IOERT boost with a cumulative incidence of telangiectasia 4.8% reported by Lemanski et al. and 4.5% in this investigation seem to be definitively not inferior to those of conventional boost irradiation [27].

After a 20-year follow-up, the EORTC study showed a significant increased incidence of fibrosis in the boost group (5.2% vs. 1.8%) [7]. Concerns had been raised that the high single dose applied as part of an IOERT potentially increases the risk of fibrosis. This was initially supported by an initial unplanned subgroup comparison conducted within the Young Boost Trial. In the Young Boost Trial, using a simultaneous integrated boost (SIB) was associated with a higher rate of fibrosis [28]. It was explained by the high single dose used in SIB. This, of course, raises the question of tolerability to IOERT, where even significantly higher single doses are used. However, there are now results from two comparative studies that have found no increased risk of fibrosis between SIB and sequential boost [29,30,31]. In a cohort irradiated with an intraoperative boost of 20 Gy, fibrosis subsequently occurred in a large number of patients (57%) after a median follow-up of 34 months [32]. Considerably better results were reported using a lower intraoperative dose of 10 Gy prior to WBRT [27]. After a follow-up of 9.1 years, 14.0% experienced grade 2 fibrosis without a report of a higher grade fibrosis. With a similar therapeutic approach, we obtained comparable fibrosis rates of 21%. Overall, IOERT seems to be beneficial regarding fibrosis despite the high single doses applied.

In general, adding a boost to whole-breast radiotherapy seems to worsen the cosmetic result. Within the EORTC trial, the 3-year panel assessment revealed that 86% of patients in the no-boost group had an excellent or good overall outcome, versus 71% of patients in the boost group (*p* = 0.0001) [33]. This is in line with the previous literature describing a very good to good cosmetic result in 65→90% of patients following a percutaneous boost [6,34,35,36]. However, modern radiotherapy techniques such as IMRT seem to achieve more favorable cosmetic outcome [37]. Since fibrosis and telangiectasia are indicators of cosmetic outcome; it had been suggested that better cosmetic results can be achieved through IOERT. Consistently, Ciabattoni et al. reported significantly superior cosmetic outcomes in the IOERT boost group in comparison to the conventional boost group according to the patients’ evaluation [8]. Unfortunately this comparison was hampered by differences in the baseline cosmetic evaluation. Consistent with our results, excellent or good outcomes were generally achieved using intraoperative procedures in 86–90% of patients, thus being comparable to the non-boost group of the EORTC study [19].

In our study, older age was shown to be a predictor of worse cosmetic outcome. Since young women have benefited most from boost irradiation in previous trials, it appears favorable that better cosmetic results are achieved in this group. Moreover, our data show that the choice of surgical procedure also has an important impact on cosmetic outcome and must be considered and documented in future analyses to better evaluate multidisciplinary outcome.

Our study is limited by the follow-up time and number of patients. We further acknowledge the retrospective study design. Another critical point is that no cosmetic examination was performed before initiation of therapy. Furthermore, the comparison to other studies is limited because different scoring systems were used.

Overall, despite these limitations, our study can show promising data on IOERT, stimulating further prospective evaluations.

## 5. Conclusions

Our investigation reports promising data on the cosmetic outcome as well as the acute and early long-term tolerability for patients treated with an IOERT boost. Our oncologic control rates are similar to the previous literature. Nevertheless, prospective randomized trials regarding the role of IOERT in comparison to other boost procedures would be desirable.

## Figures and Tables

**Table 1:** Patient and tumor characteristics.

Age (N = 139)	Mean: 54	Range: 28–43
**Risk Factors (<45 years/>2 cm/N+/G3) Present (N = 139)**		
**0**	40	28.8%
**1**	49	35.2%
**2**	31	22.3%
**3**	19	13.7%
**Laterality (N = 139)**		
**right**	67	48.2%
**left**	68	48.9%
**bilateral**	4	2.9%
**TNM tumor stage (N = 143)**		
**ypT0/yTis**	8	5.6%
**pT1a/ypT1a**	4	2.8%
**pT1b/ypT1b**	24	16.8%
**pT1c/ypT1c**	53	37.0%
**pT2/ypT2**	52	36.4%
**pT3**	2	1.4%
**TNM nodal stage (N = 143)**		
**N0**	107	74.8%
**N1**	28	19.6%
**N2**	6	4.2%
**N3**	2	1.4%
**Histopathologic type (N = 143)**		
**ductal invasive**	110	76.9%
**lobular invasive**	22	15.4%
**other**	11	7.7%
**Histologic grade (N = 143)**		
**G1**	15	10.5%
**G2**	81	56.6%
**G3**	44	30.8%
**not available (ypT0)**	3	2.1%
**Receptor status (N = 143)**		
**HR+/HER2−**	104	72.7%
**HR+/HER2+**	18	12.6%
**HR−/HER2+**	0	0.0%
**triple negative**	21	14.7%

HR: hormone receptor.

**Table 2:** Applied chemotherapy and target therapy regimens.

**Neoadjuvant Chemotherapy**			N = 19
**EC + docetaxel**	10	52.6%	
**FEC + docetaxel**	4	21.1%	
**EC + paclitacel**	3	15.8%	
**CMF + docetaxel**	1	5.3%	
**TC**	1	5.3%	
**adjuvant chemotherapy**			N = 60
**FEC + docetaxel**	30	50.0%	
**FEC + paclitaxel**	2	3.3%	
**EC + paclitaxel**	13	21.7%	
**FEC**	10	16.7%	
**TAC**	3	5.0%	
**TC**	2	3.3%	
**+trastuzumab**	21	35.0%	
**no chemotherapy**			N = 56
**not known**			N = 4

CMF: cyclophosphamide/methothrexate/5-fluorouracil; EC: epirubicin/cyclophosphamide; FEC: 5-fluorouracil/epirubicin/cyclophosphamide; TAC: docetaxel/doxorubicin/cyclophosphamide; TC: docetaxel/cyclophosphamide.

**Table 3:** Evaluation of LENT-SOMA Late toxicity.

LENT SOMA Late Toxicty (n = 66)	Grade 0 n (%)	Grade I n (%)	Grade II n (%)	Grade III+ n (%)
Pain	54 (81.8%)	11 (16.5%)	1 (1.5%)	0
Breast- edema	59 (89.4%)	6 (9.1%)	1 (1.5%)	0
Arm- edema	63 (95.4%)	3 (4.5%)	0	0
Atrophy/Retraction	61 (92.4%)	4 (6.1%)	0	1 (1.5%) Grade III
Ulcus/Necrosis	66 (100%)	0	0	0
Fibrosis	52 (78.8%)	11 (16.7%)	3 (4.5%)	0
Teleangiectasia	63 (95.4%)	3 (4.5%)	0	0
Pigmentation	51 (77.3%)	15 (22.7%)	0	0

**Table 4:** Cosmetic evaluation of all patients.

**Cosmetic Evaluation**	**Very Good** **n (%)**	**Very Good–Good** **n (%)**	**Good** **n (%)**	**Good–Moderate** **n (%)**	**Moderate** **n (%)**	**Moderate–Bad** **n (%)**	**Bad** **n (%)**	**Bad–Very Bad** **n (%)**	**Very Bad** **n (%)**
Patient	31 (47%)	0	25 (38%)	1 (2%)	6 (9%)	1 (2%)	1 (2%)	1 (2%)	0
Physician	35 (55%)	0	23 (36%)	2 (3%)	3 (5%)	0	0	1 (2%)	0
**Cosmetic** **Evaluation** **Harvard Score**	**Excellent** **n (%)**	**Good** **n (%)**		**Fair** **n (%)**			**Poor** **n (%)**		
Patient	31 (46.9%)	25 (37.9%)		8 (12.1%)			2 (3.0%)		
Physician	35 (53.0%)	23 (34.8%)		5 (7.6%)			1 (1.5%)		

**Table 5:** Characteristics of all patients and their oncological outcome.

Oncological Endpoint (n = 133)	n (%)
Local Recurrence	4 (3.0%)
Infield	0 (0%)
ipsilateral Breast	
Elsewhere ipsilateral Breast	4 (3.0%)
CBC	2 (1.5%)
Distant Metastasis	3 (2.3%)
Death	7 (5.3%)
Breast Cancer Death	4 (3.0%)
Non Breast Cancer Death	3 (2.3%)

## Data Availability

All data and materials can be accessed in compliance with the data protection regulation via C.M.
